# Patterns of sexual size dimorphism in stingless bees: Testing Rensch’s rule and potential causes in highly eusocial bees (Hymenoptera: Apidae, Meliponini)

**DOI:** 10.1002/ece3.4935

**Published:** 2019-02-05

**Authors:** José Javier G. Quezada‐Euán, Salomón Sanabria‐Urbán, Corey Smith, Raúl Cueva del Castillo

**Affiliations:** ^1^ Departamento de Apicultura Tropical, Campus de Ciencias Biológicas y Agropecuarias Universidad Autónoma de Yucatán Mérida México; ^2^ UBIPRO, Lab. de Ecología, FES Iztacala Universidad Nacional Autónoma de México Mexico City Mexico; ^3^ Invertebrate Division American Museum of Natural History New York City New York

**Keywords:** body size, caste determination, *Melipona*, Rensch’s rule, sexual dimorphism

## Abstract

Eusocial insects offer a unique opportunity to analyze the evolution of body size differences between sexes in relation to social environment. The workers, being sterile females, are not subject to selection for reproductive function providing a natural control for parsing the effects of selection on reproductive function (i.e., sexual and fecundity selection) from other kinds of natural selection. Patterns of sexual size dimorphism (SSD) and testing of Rensch's rule controlling for phylogenetic effects were analyzed in the Meliponini or stingless bees. Theory predicts that queens may exhibit higher selection for fecundity in eusocial taxa, but contrary to this, we found mixed patterns of SSD in Meliponini. Non‐*Melipona* species generally have a female‐biased SSD, while all analyzed species of *Melipona *showed a male‐biased SSD, indicating that the direction and magnitude of the selective pressures do not operate in the same way for all members of this taxon. The phylogenetic regressions revealed that the rate of divergence has not differed between the two castes of females and the males, that is, stingless bees do not seem to follow Rensch's rule (a slope >1), adding this highly eusocial taxon to the various solitary insect taxa not conforming with it. Noteworthy, when *Melipona* was removed from the analysis, the phylogenetic regressions for the thorax width of males on queens had a slope significantly smaller than 1, suggesting that the evolutionary divergence has been larger in queens than males, and could be explained by stronger selection on female fecundity only in non‐*Melipona* species. Our results in the stingless bees question the classical explanation of female‐biased SSD via fecundity and provide a first evidence of a more complex determination of SSD in highly eusocial species. We suggest that in highly eusocial taxa, additional selection mechanisms, possibly related to individual and colonial interests, could influence the evolution of environmentally determined traits such as body size.

## 1 INTRODUCTION

Sexual size dimorphism (SSD) is a widespread phenomenon across the animal and plant kingdoms. It is generally accepted that patterns of SSD result from the selective forces acting on male versus female body size in relation to their different reproductive roles (Andersson, 1994; Tammaru, Esperk, Ivanov, & Teder, 2010; Teder, 2014). That is, selection acts for high fecundity in females, while sexual selection to monopolize females drives body size increase in males (Blanckenhorn, 2000; Webb & Freckleton, 2007). However, the ultimate causes of SSD have remained elusive and it has been difficult to determine the relative impact of the interplay between natural and sexual selection in the evolution of SSD. In most invertebrates, females are the larger sex (Stillwell, Blanckenhorn, Teder, Davidowitz, & Fox, 2010; Teder, 2014; Teder & Tammaru, 2005), and in insects, it has been estimated that between 72% and 95% of species within each order exhibit female‐biased SSD suggesting that fecundity selection may be the major driving force acting on SSD in most orders (Stillwell et al., 2010).

The evolution of SSD has remained understudied in social insects. However, highly eusocial insects are an interesting model to analyze the relative importance of fecundity and sexual selection, because in this group of organisms the females are divided into reproductive queen and (mostly) sterile worker castes (Michener, 1974; Wilson, 1971). As workers perform nest duties, queens are presumably released from the constraints of foraging and nest building, and might more readily respond to fecundity selection (Shreeves & Field, 2008). Although studies of highly eusocial taxa are limited, it is expected that queens should exhibit consistently larger sizes than males (Boomsma, Baer, & Heinze, 2005). This may be particularly reinforced in highly eusocial bees in which males do not fight to monopolize groups of females and engage in scramble competition for sexual partners (Beani, Dessì‐Fulgheri, Cappa, & Toth, 2014; Paxton, 2005).

In many animal taxa, the allometry of body size among sexes varies in accordance with the pattern of SSD, it increases when males are the larger sex, but decreases with body size when females are larger than males (Abouheif & Fairbairn, 1997; Fairbairn, 1997; Rensch, 1950). These trends are explained by greater evolutionary divergence and plasticity in male size, compared with female size, a pattern known as Rensch's rule (Fairbairn, 1997; Rensch, 1950). Although there are several explanations for Rensch's rule, this allometric trend is usually attributed to sexual selection acting on male body size (Fairbairn, Blanckenhorn, & Székely, 2007; Stillwell et al., 2010). High levels of competition among males are consistent with Rensch's rule (Dale et al., 2007). The converse trend, where female size varies more than male size, is less common, but seems to be the result of strong fecundity selection acting on females (Blanckenhorn et al., 2007; Foellmer & Moya‐Larano, 2007; Webb & Freckleton, 2007) or on small males that perform aerial displays (Dale et al., 2007).

Interestingly, in insects, allometric patterns have not always conformed to Rensch's rule. Notably, in solitary Hymenoptera a consistent opposite trend has been found, with variance in female size tending to be greater than variance in male size (Blanckenhorn et al., 2007; Fairbairn et al., 2007). Similar to solitary species, a first macroevolutionary study of SSD and Rensch's rule on the primitively social corbiculate bumblebees (Tribe Bombini) revealed a predominantly female‐biased SSD but allometric patterns not conforming to Rensch's rule (Cueva del Castillo & Fairbairn, 2012). Therefore, fecundity selection seemed stronger compared with sexual selection in corbiculate eusocial bees (Stubblefield & Seger, 1994; Boomsma et al., 2005). Surprisingly, in a recent intraspecific study, Medina et al. (2016) found moderately male‐biased SSD in the highly eusocial stingless bee *Melipona beecheii* (Tribe Meliponini) and the honeybee *Apis mellifera *(Tribe Apini), and only little difference between males and females in *Euglossa mellifera*, an eusocial primitive species of the Tribe Euglossini. Thus, contrasting differences in the patterns of SSD have been revealed among the four Tribes comprising the corbiculate bees, possibly related to the level of sociality in this group (Medina et al., 2016). However, an extensive macroevolutionary analysis in the highly eusocial bees with which to identify proximate and ultimate explanations is lacking.

We used the stingless bees (Tribe Meliponini) as a model to test hypotheses related to body size and allometry in the highly eusocial bees. The Meliponini are a clade of exclusive highly eusocial species living in colonies with morphologically different queen and workers (Sakagami, 1982). The stingless bees offer the opportunity to better analyze body size patterns because of the high diversity of species (ca. 500 with pantropical distribution; Rasmussen & Cameron, 2010), in contrast with the only other group of highly eusocial bees, the Apini, in which only ten species have been recognized (Oldroyd & Wongsiri, 2006). However, in contrast with the Apini, the biology of most species of stingless bees remains unstudied.

We analyzed the evolutionary divergence in body size and sexual size dimorphism among stingless bees compared with primitively social and solitary closer relatives using a series of allometric predictions tested by Cueva del Castillo and Fairbairn (2012) in bumblebees. After controlling by phylogenetic effects, we expected that our results would reflect that selection for female fertility would act equally in all species of stingless bees and that female‐biased SSD would be the norm in this clade. We also expected that selection to act more strongly on queens and males than on workers because workers, being sterile, experience selection only indirectly through their effects on colony success (Kovacs, Hoffman, Marriner, & Goodisman, 2010; Linksvayer & Wade, 2009). Thus, a comparison of the evolutionary divergence of queens and males to that of workers should reveal the effects of selection on reproductive function (i.e., fecundity and sexual selection).

If the divergence caused by fecundity selection on queens’ size has been greater than sexual selection on males, the regression of male size on queen size should have a slope below 1, not following Rensch's rule. Otherwise, a slope >1 would indicate that evolutionary divergence caused by sexual selection on males has exceeded fecundity selection on queens. In addition, if the effect of sexual selection on males exceeds that of natural selection on workers, we predict that the regression of male against worker size will have a slope >1. Because it is expected that queens are under strong fecundity selection, we predict that the regression of queen size on worker size selection for foraging and brood care should have a slope >1.

## 2 METHODS

For the analyses, we selected an estimator of body size at emergence of the different individuals. For this, we used intertegular distance, a measure currently accepted as standard of body size in different bee taxa, that does not seem to change with age (Bullock, 1999; Greenleaf, Williams, Winfree, & Kremen, 2007).

The data on queens, males, and workers used in this study were directly measured on specimens from collections at Universidad Autónoma de Yucatán (J Quezada‐Euán, 12 species), the American Museum of Natural History (Corey Smith, six species), Universidad Nacional de Colombia (Guiomar Nates‐Parra, three species), and the University of Western Sydney (Megan Halcroft, five species). Additionally, we searched published data on size of different species of stingless bees by executing a search on Google Scholar using the terms “body size,” “intertegular width,” and “thorax width.” Google Scholar was used as the search engine because it usually provides full‐text versions of published papers. Data on fifteen more species were obtained from this search. In addition, data from females and males of solitary corbiculate species *Euglossa imperialis* and *Exaerete smaragdina* collected in Los Tuxtlas, Veracruz, (Cueva del Castillo, unpublished data) were included in the study.

### 2.1 Phylogenetic reconstruction

To remove the phylogenetic effects from the analysis of data, we built a phylogeny of Meliponini species mainly based on the genetic information available on the GenBank. This genetic information involved partial sequences of two mitochondrial loci (cytochrome oxidase subunit I, COI; and 16S ribosomal RNA, 16S) and four nuclear loci (arginine kinase, ArgK; elongation factor 1 alpha, EF‐1α; long‐wavelength rhodopsin, LWR; and 28S ribosomal RNA, 28S) that were used in previous phylogenetic studies in stingless bees (Françoso & Arias, 2013; Halcroft et al., 2016; May‐Itza, Quezada‐Euán, Medina, Enríquez, & De la Rúa, 2010; Ramírez et al., 2010; Rasmussen & Cameron, 2010; Ruiz, May‐Itza, Quezada‐Euán, & De la Rúa, 2013). Particularly, COI sequences for three Meliponini species (*Frieseomelitta nigra*, *Lestrimelitta niitkib*, and *Partamona bilineata*) were provided by the Canadian Barcode of Life Network. In order to match the species with the data in the molecular sources, we first homologated the taxonomic information found in the literature search with the updated revision by Camargo and Pedro (2007) and Rasmussen and Cameron (2010). We also retrieved from GenBank genetic information of five corbiculate bee species (*Apis dorsata*, *Bombus terrestris*, *Euglosa imperialis*, *Eulaema boliviensis*, and *Exaerete smaragdina*) that were used as outgroups for the phylogenetic analysis as in previous studies. We provide the accession numbers of the GenBank's used sequences on the Supporting Information Table S1.

We aligned the nucleotide sequences of each locus using the algorithm implemented in MUSCLE (Edgar, 2004). We removed the ending sequence fragments that did not overlap with at least 80% of the species in each aligned dataset. The final number of aligned positions for each dataset was as follows: 1200 for COI, 606 for 16S, 1003 for ArgK, 874 for EF‐1α, 605 for LWR, and 865 for 28S. We then used MESQUITE 3.51 (Maddison & Maddison, 2015) to concatenate the sequences of each gene and to make the final total evidence matrix for all genes, which comprised 5034 aligned positions and 258 terminals.

We initially partitioned our concatenated matrix in nine partitions considering the coding (COI) and noncoding mitochondrial loci (16S and 28S), as well as the intron and exon regions of the nuclear coding loci (ArgK‐introns, ArgK‐exons, EF‐1α‐introns, EF‐1α‐exons, LWR‐introns, and LWR‐exons). We then estimated the best partition scheme and nucleotide substitution models for each partition using the greedy algorithm implemented in PartitionFinder 1.1.1 (Lanfear, Calcott, Kainer, Mayer, & Stamatakis, 2014). According to this analysis, we subdivided the final concatenated matrix in seven partitions (P1–P7) that involved the following loci: P1 (16S), P2 (28S and LWR‐exons), P3 (COI), P4 (ArgK‐exons), P5 (ArgK‐introns), P6 (EF‐1α‐exons), and P7 (EF‐1α‐introns and LWR‐introns). The best substitution models estimated for these partitions where as follows: GTR+I+G for P1, P3, and P7; K80+I+G for P2 and P4; and HKY for P5 and P6.

We conducted a concatenated Bayesian inference (BI) analysis in MrBayes 3.2.6 (Ronquist et al., 2012) with the total evidence matrix by applying the specific substitution model estimated for each partition individually. The BI analysis consisted of four independent runs, each with 20,000,000 generations and four chains, sampling every 2,000 generations. We used default priors for other parameters in the analysis. We assessed parameter convergence and proper mixing of independent runs using TRACER 1.6 (Rambaut, Drummond, Xie, Baele, & Suchard, 2018). All parameter values sampled during the MCMC of the analysis resulted in ESS values >200. We discarded 25% of the samples obtained prior to stability as burn‐in to obtain a final consensus phylogeny. We used this consensus phylogeny for our posterior comparative analysis considering it was largely congruent with the most robust phylogenies estimated in the group at present (Ramírez et al., 2010; Rasmussen & Cameron, 2010).

### 2.2 Comparative analyses

To convert branch lengths of the consensus phylogeny to units of time, we conducted a divergence time analysis in BEAST v1.8.4 (Drummond & Rambaut, 2007). For this analysis, we used a relaxed clock uncorrelated lognormal method and we assumed a birth–death speciation process for the tree prior to using the topology of our consensus phylogeny. We also applied the same partition scheme and substitution models as in the MrBayes analysis. For the age parameters of the root node (Corbiculates) and the crown group (Meliponini), we assumed normal prior distributions with means and standard deviations as follows: 78 ± 5 Mya and 54 ± 3 Mya, respectively. These two age calibration points were obtained from the most robust divergence study of bees’ lineages at present (Cardinal & Danforth, 2013). MCMC searches were run for 100,000,000 generations, sampling every 10,000 generations. We performed three independent analysis in CIPRES (Miller, Pfeiffer, & Schwartz, 2010). We assessed parameter convergence and proper mixing of independent analysis using TRACER 1.6 (Rambaut, Drummond, Xie, Baele, & Suchard, 2018). All parameter values sampled during the MCMC of the analyses resulted in ESS values >200. We discarded 25% of the samples obtained prior to stability as burn‐in to obtain the chronogram. We used this resulting ultrametric phylogeny to perform all comparative analysis, pruning the species for which we did not obtain morphologic data with the R (version 3.1.3; R Core Team, 2015) package “ape” (Paradis, Claude, & Strimmer, 2004). A data file comprising the total evidence matrix of DNA alignments, the consensus BI phylogeny and the chronogram that resulted from the above described analyses is available on the TreeBASE (accession number: 23779).

### 2.3 Ancestral reconstruction of SSD

In order to explore the evolutionary trends in body size and SSD, we used the thorax width of males and queens to build a SSDi index following the Lovich and Gibbons (1992) criteria. This index expresses SSD as [(length of larger sex/length of smaller sex) − 1]. For convention, the SSDi is arbitrarily changed to negative when males are the larger sex and positive when females are the larger sex (Cox, Butler, & John‐Alder, 2007). Then, we performed an ancestral character reconstruction following Revell (2013) and considering the SSDi values estimated from Meliponini species, as well as from other corbiculate bees species (*A. dorsata*,* B. terrestris*,* E. imperialis*, and *E. smaragdina*). This method estimates the maximum likelihood value for internal nodes and then interpolates the states along the branches of the phylogenetic tree (see for details Revell, 2013, 2014). For the reconstruction and visualization of ancestral state reconstruction of SDI, we used the R package “Phytools” (Revell, 2012).

### 2.4 Rensch's rule

Rensch's rule predicts that the slope of a regression of male body size on queen and workers body size will be steeper than 1. To test these predictions in Meliponini species, we used the phylogenetic independent contrasts method (PIC method; Felsenstein, 1985), as implemented by the package “Caper” (Orme et al., 2013) in R (ver. 3.0.1; R Development Core Team 2013) to control for the phylogenetic nonindependence of species (Harvey & Pagel, 1991). We examined the studentized residuals for outliers >|±3|, but found none in our dataset. Ultimately, we tested the allometric relationship between independent contrasts of log10 male thorax width on log10 queen and worker thorax width, and the allometric relationship between independent contrasts of log10 queen thorax width on the log10 worker thorax width by fitting major axis regression using the R package “smatr” (Warton, Duursma, Falster, & Taskinen, 2012). Females and males of the other social and solitary corbiculate species were included in the regression of males on queens. Major axis regression offers an accurate approach to test the null hypothesis of isometry (h0: *β* = 1), because both variables were measured on the same scale and residual variance is minimized in both variables (Warton, Duursma, Falster, & Taskinen, 2012). Because species of the genus *Melipona* showed a different trend in the evolution of SSD than the other bees (see below), we performed another phylogenetic independent contrasts analyses excluding this genus in order to evaluate their relative weight in the evolutionary divergence of female and male size in the Tribe.

## 3 RESULTS

### 3.1 Ancestral reconstruction of SSD

**Figure 1:**
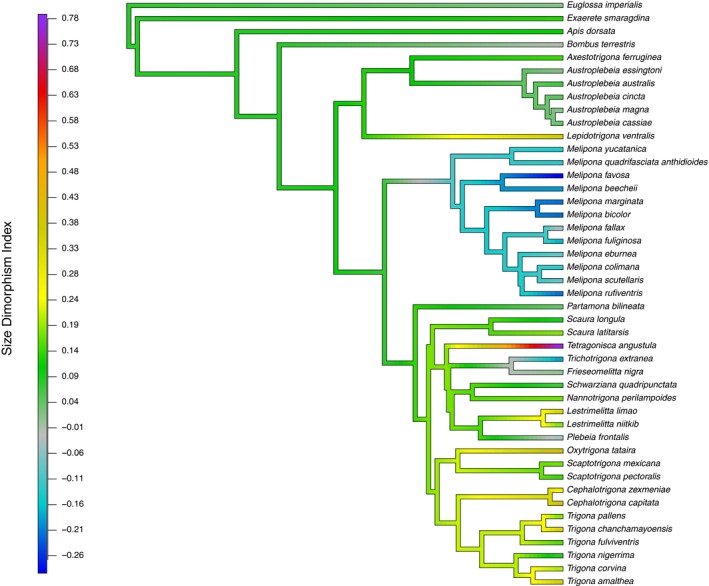
Maximum likelihood ancestral reconstruction of SSDi for 37 social and solitary bee taxa performed in R package “phytools” (Revell, 2012). For the analysis, we used the ultrametric phylogeny and the values of SDI estimated for each species. The values in the color ramp represent the range of SSDi registered for the study species. Negative values indicate male‐biased SSD (blue to gray) and positive values female‐biased SSD (green to violet). SSD: sexual size dimorphism.

We found a continuum from a moderate SSD bias to males to a SSD bias to females (Figure 1). In 23 species (57%), there is a female‐biased SSD, in six species, males and females showed similar body sizes (*Austroplebeia magna*, *Austroplebeia cassia*,* Austroplebeia essingtoni*,* F. nigra*,* Melipona fallax*, and *Plebeia frontalis*), and 32% (12 species) showed some degree of male‐biased SSD, particularly those of *Melipona* (Table 1, Supporting Information Figure S1). Considering that solitary species are predominantly female‐biased SSD, a moderate female‐biased dimorphism or no dimorphism could be the ancestral condition in the corbiculate bees. In our sample, we found that male‐biased SSD possibly evolved independently two times, in *Melipona* and* Trichotrigona extranea *(Figure 1). Interestingly, the level of SSD seems not related to the body size of the species. Thus, *M. beecheii *and *Melipona fuliginosa *show similar levels of SSD bias to males (≈−0.15; Table 1), but the mean thorax size of the last species is larger ($\overset{¯}{X}$ ≈ 3.63 mm) than *M. beecheii* ($\overset{¯}{X}$ ≈ 2.36 mm) (Table 1). Similarly, *L. niitkib* and *Nannotrigona perilampoides* have similar levels of SSD bias to females (≈0.19). However, the first species is around 30% larger than the latter. Moreover, species like *Trigona amalthea*,* Trigona chanchamayoensis*, and *Cephalotrigona capitata *with a relatively large body size ($\overset{¯}{X}$ ≈ 2.35, 2.38, and 3.19 mm, respectively) show high levels of SSD bias to females (>0.35), than the average for the other non‐*Melipona* species (Thorax width $\overset{¯}{X}$ ≈ 0.18), and the smallest species (*Austroplebeia *group, *P. frontalis*, *Scaura longula*, and *Tetragonula iridipennis*) show small levels of SSD bias to both females and males (Table 1).

**Table 1:** Intertegular width values for workers, males, and queens from 37 social and solitary bee species, SSD_i_: Lovich and Gibbons Sexual Size Dimorphism index estimated for queen and males (negative values indicate when males are the larger sex), and average thorax width per species (considering just reproductive individuals)

Species	Thorax width (mm)				Species mean thorax size (mm)
	Worker	Male	Queen	SSD_i_	
*Austroplebeia australis* d	1.31	1.31	1.43	0.091	1.37
*Austroplebeia cassiae* d	1.36	1.37	1.45	0.058	1.41
*Austroplebeia cincta* d	1.11	1.15	1.23	0.070	1.19
*Austroplebeia essingtoni* d	1.10	1.13	1.17	0.035	1.15
*Austroplebeia magna* d	1.33	1.35	1.39	0.030	1.37
*Axestotrigona ferruginea* c	1.925	2.00	2.32	0.160	2.16
*Bombus terrestris* k	4.77	7.76	7.86	0.013	7.81
*Cephalotrigona capitata* b	2.88	2.63	3.75	0.425	3.19
*Cephalotrigona zexmeniae* m	2.081	1.925	2.468	0.282	2.196
*Euglossa imperialis* n		5.38	5.61	0.043	5.50
*Exaerete smaragdina* n		7.05	8.11	0.150	7.58
*Frieseomelitta nigra* m	1.227	1.307	1.352	0.034	1.329
*Lepidotrigona ventralis* f	1.44	1.48	2.05	0.385	1.765
*Lestrimelitta limao* b	2.00	2.00	2.75	0.375	2.375
*Lestrimelitta niitkib* m	1.493	1.584	1.89	0.191	1.737
*Melipona beecheii* l	2.528	2.556	2.158	−0.155	2.357
*Melipona bicolor* b	3.875	4.00	3.25	−0.188	3.625
*Melipona colimana* m	2.894	2.985	2.699	−0.095	2.842
*Melipona eburnea* e	4.89	4.708	4.409	−0.063	4.558
*Melipona fallax* h	3.43	3.53	3.48	−0.014	3.51
*Melipona favosa* i	2.633	2.675	1.968	−0.264	2.321
*Melipona fuliginosa* h	3.68	3.92	3.33	−0.151	3.625
*Melipona marginata* i	2.299	2.307	1.86	−0.193	2.083
*Melipona quadrifasciata* i	2.888	2.884	2.61	−0.095	2.747
*Melipona rufiventris* a	3.75	4.00	3.25	−0.188	3.63
*Melipona scutellaris* j	3.01	2.87	2.71	−0.056	2.79
*Melipona yucatanica* m	2.235	2.245	1.97	−0.11	2.107
*Nannotrigona perilampoides* m	1.309	1.224	1.453	0.187	1.338
*Oxytrigona tataira* i	1.526	1.522	2.16	0.424	1.841
*Partamona bilineata* m	1.589	1.561	1.655	0.060	1.608
*Plebeia frontalis* m	1.02	1.018	0.993	−0.024	1.00
*Scaptotrigona mexicana* m	1.453	1.524	1.82	0.194	1.672
*Scaptotrigona pectoralis* m	1.613	1.582	1.831	0.157	1.706
*Scaura latitarsis* b	1.25	1.25	1.50	0.200	1.375
*Scaura longula* b	1.50	1.50	1.66	0.107	1.58
*Schwarziana quadripunctata* i	1.748	1.865	2.039	0.093	1.952
*Tetragonisca angustula* e	1.019	1.061	1.886	0.777	1.473
*Trichotrigona extranea* g	NA	1.48	1.25	−0.155	1.365
*Trigona amalthea* e	2.218	1.982	2.729	0.376	2.355
*Trigona chanchamayoensis* b	1.88	2.00	2.75	0.375	2.375
*Trigona corvina* b	2.13	2.25	2.75	0.222	2.50
*Trigona fulviventris* m	1.429	1.606	1.871	0.165	1.738
*Trigona nigerrima* b	2.38	2.50	2.75	0.100	2.625
*Trigona pallens* i	1.416	1.722	2.047	0.188	1.884

a Schwarz (1932).

b Schwarz (1948).

c Darchen (1971).

d Halcroft (unpublished data).

e Nates‐Parra (unpublished data).

f Sung, Yamane, Ho, Wu, and Chen (2004).

g Camargo and Pedro (2007).

h Camargo and Pedro (2008).

i Smith (unpublished data).

j de Araujo‐Alves (2010).

k Cueva del Castillo, Sanabria‐Urbán, and Serrano‐Meneses (2015).

l Medina et al. (2016).

m Quezada‐Euán (unpublished data).

n Cueva del Castillo (unpublished data).

### 3.2 Rensch's rule

**Figure 2:**
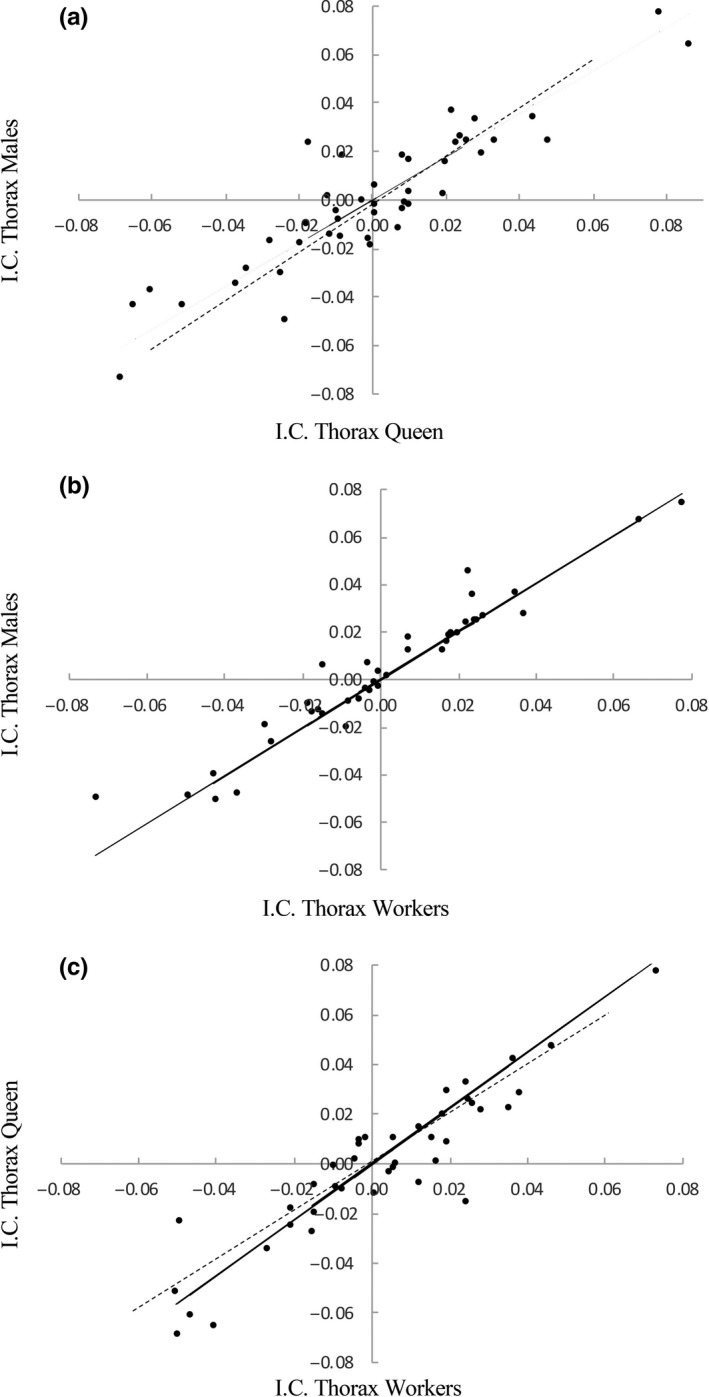
Allometric major axis regressions of independent contrasts (IC) of (a) males’ on queens’, (b) males’ on workers’, and (c) queens’ on workers’ thorax width of stingless bees. Dashed lines indicate isometry (*β* = 1)

**Figure 3:**
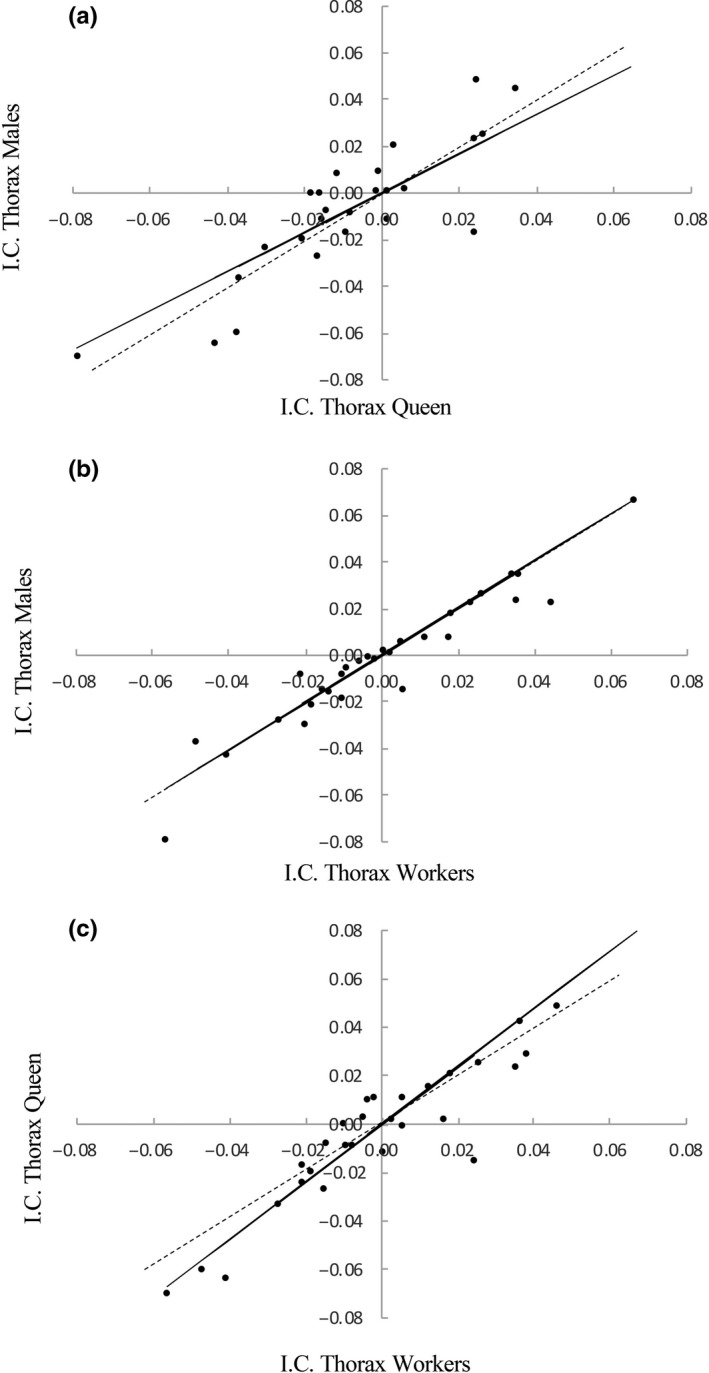
Allometric major axis regressions of independent contrasts (IC) after removing *Melipona* species of (a) males’ on queens’, (b) males’ on workers’, and (c) queens’ on workers’ thorax width of stingless bees. Dashed lines indicate isometry (*β* = 1)

After controlling for phylogenetic nonindependence among of the species studied, the results of the major axis regression of independent contrasts indicated strong coevolution between queens and males (*r* ^2^ = 0.82; *df* = 42, *p* < 0.0001), between queens and workers (*r* ^2^ = 0.86; *df* = 39, *p* < 0.0001), and between males and workers (*r* ^2^ = 0.93; *df* = 39, *p* < 0.0001; Figure 2). However, none of the regressions showed a slope significantly different from 1 (males on workers: *β* = 1.01, *p* = 0.89; queens on workers: *β* = 1.12, *p* = 0.09; and males on queens: *β* = 0.89, *p* = 0.12). Thus, it seems that evolutionary divergence has been similar in males and the female sexual and sterile castes. Nonetheless, after removing *Melipona* species from the analyses, we found that the slope of males on queens was significant and smaller than 1 (*β* = 0.84, *p* = 0.04; Figure 3) and the slope of the regressions of queen on workers (*β* = 1.19, *p* = 0.03) was significant and stepper than 1, suggesting that queen size has diverged more than male and worker size in non‐*Melipona* species, even though males on workers (*β* = 1.01, *p* = 0.91) remain no significant.

## 4 DISCUSSION

Contrary to expectations, our results quantitatively confirm the existence of two different patterns of SSD in stingless bees, revealing the Meliponini as a taxon with mixed SSD. Nevertheless, compared to solitary species moderate levels of SSD were found. In addition, no evidence of hyperallometry was evident when the size of males was regressed on queens, making Meliponini a first clade of highly eusocial insects in which it is shown that Rensch's rule does not hold (Abouheif & Fairbairn, 1997; Blanckenhorn et al., 2007; Cueva del Castillo & Fairbairn, 2012; Fairbairn, 1997; Webb & Freckleton, 2007). Perhaps due to the independent evolution of differences in size and SSD bias to females and males (Figures 2 and 3), we did not detect significant departures from isometry for our phylogenetically controlled regressions for the thorax width of males on queens, males on workers, and queens on workers. Nonetheless, when we removed *Melipona* from the analysis, the phylogenetically controlled regressions for the thorax width of males on queens have a slope significantly smaller than 1, whereas the regression of queens on workers has a slope significantly stepper than 1, suggesting that similar to other eusocial species the magnitude of evolutionary divergence has been larger in queen than males, and could be explained by stronger selection on female fecundity. This strong selection of queen's fecundity also can explain the size divergence between queens and the sterile workers in non‐*Melipona* species (Fairbairn et al., 2007).

Apparently, independent evolution of differences in size and SSD bias to queens and males suggests that the interplay between natural and sexual selection has operated in different ways in Meliponini, resulting in two patterns of SSD, male‐ and female‐biased, which also suggest that as in other animals, the direction and magnitude of the selective pressures do not operate in the same way for all members of this taxon (Colwell, 2000; Kratochvíl & Frynta, 2007; Serrano‐Meneses, Cordoba‐Aguilar, Azpilicueta‐Amorín, González‐Soriano, & Székely, 2008). The reproductive behavior of stingless bees remains largely unknown (Quezada‐Euán, 2018). The lack of sufficient empirical data prevents an accurate explanation of the evolutionary meaning and reproductive implications of the opposite patterns of SSD found in this taxon. One possible explanation for the male‐biased SSD found in *Melipona *species and *T. extranea* could be sexual selection acting on males’ body size. Even though there are few studies of sexual selection in social insects (Berg, Koeniger, Koeniger, & Fuchs, 1997; Boomsma & Ratnieks, 1996), there is no reason to discard its importance in the evolution of male traits (Baer, 2005; Boomsma et al., 2005) and SSD bias to males in social insects, especially when it is well known that males from eusocial bees usually engage in scramble competition during mating events (Paxton, 2005). Nevertheless, differences in SSD in stingless bees could also be related to selection on the size of queens rather than males. Indeed, it has been suggested that reproductive females when released from the burden imposed by the collection of food and construction of nests can experience more flexibility in their development (Medina et al., 2016). Cuckoo bees, for instance, show a tendency toward less female‐biased SSD compared to nest‐building species (Shreeves & Field, 2008). Likewise, in *M. beecheii*, Medina et al. (2016) found that weight at emergence varied widely among individual queens (up to fourfold) compared with males (less than twofold).

A proximate cause to explain size differences among sexes in solitary insects is the difference in development time of males and females (Teder, 2014). Differences in development time of queens relative to males could also extend to explain differences among genera in Meliponini. Empirical data indicate that in *Melipona* males, workers and queens receive similar amounts of food as larvae and pass through the same stages of development, but the pupae stage of queens is shorter than in males and workers (Jarau et al., 2010; Macías‐Macías & Quezada‐Euán, 2015; Moo‐Valle, Quezada‐Euán, Canto‐Martín … González‐Acereto, 2004; Quezada‐Euán, 2018; Quezada‐Euán et al., 2011). Such accelerated rate of development can explain why the queens in *Melipona *species are comparatively smaller than the queens of non‐*Melipona,* in which queens take longer to develop than males and workers, probably as a consequence of the excess food they receive as larvae. In support for this argument, in non‐*Melipona* species, female larvae receiving less food can also become queens, but of minute size which also have reduced development times (Morales, 2018). In *Melipona, *female larvae become queens not only by food but also by a possible genetic mechanism (Jarau et al., 2010; Kerr, 1948).

Interestingly, *Melipona* colonies produce excess queens that are eliminated by the workers (Wenseleers, Hart, Ratnieks, & Quezada‐Euán, 2004). Such apparently unnecessary production of surplus queens in *Melipona* has been explained as result from the selfish behavior of female larvae attempting to evade a worker fate with less reproductive advantages (Ratnieks, 2001; Ratnieks & Wenseleers, 2005). Perhaps, a fast rate of development (and the resulting small size) is a result of the competition among the many queens that are produced at one time in *Melipona, *but not in the other genera of stingless bees, which produce substantially less queens. Queens relieved from the burden of nest construction, and provision can trade traits requiring large body size for faster development that would result in smaller size. However, this would be adaptive only if queen fitness may not suffer as a consequence of reduced size up to one point. Indirect empirical evidence from non‐*Melipona* species suggests that minute queens (presumably experiencing fast development rates) are capable of heading colonies, which seems to confirm this hypothesis (Imperatriz‐Fonseca & Zucchi, 1995; Ribeiro, Wenseleers, Santos Filho, & Alves, 2006).

Our results in the stingless bees provide a first evidence of additional evolutionary forces that may influence SSD in highly eusocial species. We suggest that in highly eusocial taxa, a trade‐off between individual and colonial interests, in addition to sexual and fecundity selection, could influence the evolution of environmentally determined traits such as body size. The collection of more empirical data on the relative size of males and females in highly eusocial insects is necessary to test such hypotheses. Studies comparing body size variation of males and females within and across regions are also needed to analyze environmental effects that may determine male and female fitness in highly eusocial insects.

## CONFLICT OF INTEREST

None declared.

## AUTHOR CONTRIBUTIONS

J. Javier G. Quezada‐Euán collected and provided part of the data required for this work and contributed in the process to write the paper. Corey Smith collected and provided part of the data required for this paper. Salomón Sanabria‐Urbán performed the phylogenetic reconstructions, performed part of the comparative analyses, and contributed in the process to write the paper. Raul Cueva del Castillo performed part of the comparative analyses and contributed to writing of the paper.

## Supporting information

FigS1

## Data Availability

The genetic and morphological information used in this study was mainly obtained from previously published studies which are cited throughout the article. The GenBank accession numbers and source references of all the used gene sequences are provided in the Supplementary Information Table S1. The total evidence datasets used for our analyses, including the obtained DNA alignments, consensus BI phylogeny and Chronogram, are also available on the TreeBASE with the following accession number: 23779.

## References

[ece34935-bib-0001] Abouheif, E. , & Fairbairn, D. J. (1997). A comparative analysis of allometry for sexual size dimorphism: Assessing Rensch’s rule. The American Naturalist, 149, 540–562. 10.1086/286004

[ece34935-bib-0002] Andersson, M. (1994). Sexual selection. Princeton, NJ: Princeton University Press.

[ece34935-bib-0003] Baer, B. (2005). Sexual selection in Apis bees. Apidologie, 36, 187–200.

[ece34935-bib-0004] Beani, L. , Dessì‐Fulgheri, F. , Cappa, F. , & Toth, A. (2014). The trap of sex in social insects: From the female to the male perspective. Neuroscience and Biobehavioral Reviews, 46, 519–533. 10.1016/j.neubiorev.2014.09.014 25280909

[ece34935-bib-0005] Berg, S. , Koeniger, N. , Koeniger, G. , & Fuchs, S. (1997). Body size and reproductive success of drones (*Apis mellifera* L). Apidologie, 28, 449–460. 10.1051/apido:19970611

[ece34935-bib-0006] Blanckenhorn, W. U. (2000). The evolution of body size: What keeps organisms small? The Quarterly Review of Biology, 75, 385–407. 11125698 10.1086/393620

[ece34935-bib-0007] Blanckenhorn, W. U. , Dixon, A. F. G. , Fairbairn, D. J. , Foellmer, M. W. , Gibert, P. , van der Linde, K. , … Wiklund, C. (2007). Proximate causes of Rensch’s rule: Does sexual size dimorphism in arthropods result from sex differences in development time? The American Naturalist, 169, 245–257. 10.1086/510597 17211807

[ece34935-bib-0008] Boomsma, J. J. , Baer, B. , & Heinze, J. (2005). The evolution of male traits in social insects. Annual Review of Entomology, 50, 395–420. 10.1146/annurev.ento.50.071803.130416 15822204

[ece34935-bib-0009] Boomsma, J. J. , & Ratnieks, F. L. W. (1996). Paternity in eusocial Hymenoptera. Philosophical Transactions of the Royal Society B, 351, 947–975.

[ece34935-bib-0010] Bullock, S. H. (1999). Relationships among body size, wing size and mass in bees from a tropical dry forest in México. Journal of the Kansas Entomological Society, 72, 426–439.

[ece34935-bib-0011] Camargo, J. M. F. , & Pedro, S. R. M. (2007) *Meliponini Lepeletier* , 1836. Retrieved from http://www.moure.cria.org.br/catalogue

[ece34935-bib-0012] Camargo, J. M. F. , & Pedro, S. R. M. (2008). Revisão das espécies de *Melipona* do grupo fuliginosa (Hymenoptera, Apoidea, Apidae, Meliponini). Revista Brasileira De Entomologia, 52, 411–427. 10.1590/S0085-56262008000300014

[ece34935-bib-0013] Colwell, R. K. (2000). Rensch’s rule crosses the line: Convergent allometry of sexual size dimorphism in hummingbirds and flower mites. The American Naturalist, 156, 495–510. 10.1086/303406 29587514

[ece34935-bib-0014] Cardinal, S. , & Danforth, B. N. (2013). Bees diversified in the age of eudicots. Proceedings of the Royal Society B: Biological Sciences., 280(1755), 20122686 10.1098/rspb.2012.2686 PMC3574388 23363629

[ece34935-bib-0015] Cox, R. M. , Butler, M. A. , & John‐Alder, H. B. . (2007) The evolution of sexual size dimorphism in reptiles In Sex, size and gender roles: Evolutionary studies of sexual size dimorphism (pp. 38–49). New York, NY: Oxford University Press.

[ece34935-bib-0016] Cueva del Castillo, R. , & Fairbairn, D. J. (2012). Macroevolutionary patterns of bumblebee body size: Detecting the interplay between natural and sexual selection. Ecology and Evolution, 2, 46–57. 10.1002/ece3.65 22408725 PMC3297177

[ece34935-bib-0017] Cueva del Castillo, R. , Sanabria‐Urbán, S. , & Serrano‐Meneses, M. A. (2015). Trade‐offs in the evolution of bumblebee colony and body size: A comparative analysis. Ecology and Evolution, 5, 3914–3926. 10.1002/ece3.1659 26445652 PMC4588658

[ece34935-bib-0018] Dale, J. , Dunn, P. O. , Figuerola, J. , Lislevand, T. , Székely, T. , & Whittingham, L. A. (2007). Sexual selection explains Rensch’s rule of allometry for sexual size dimorphism. Proceedings of the Royal Society B: Biological Sciences, 274(1628), 2971–2979. 10.1098/rspb.2007.1043 PMC2211517 17878139

[ece34935-bib-0019] Darchen, R. (1971). *Trigona* (Axestotrigona) *oyani* Darchen (Apidae, Trigoninae), une nouvelle espèce d’abeille africaine‐description du nid inclus dans une fourmilière. Biologia Gabonica, 7, 407–421.

[ece34935-bib-0020] de Araujo‐Alves, D. (2010). *Estratégias Reprodutivas Em Melipona. Com Ênfase Em Pequenas Populações de Melipona scutellaris (Apidae, Meliponini)* . São Paulo: Universidade de São Paulo.

[ece34935-bib-0021] Drummond, A. J. , & Rambaut, A. (2007). BEAST: Bayesian evolutionary analysis by sampling trees. BMC Evolutionary Biology, 7, 214 10.1186/1471-2148-7-214 17996036 PMC2247476

[ece34935-bib-0022] Edgar, R. C. (2004). MUSCLE: A multiple sequence alignment method with reduced time and space complexity. BMC Bioinformatics, 5, 113. 15318951 10.1186/1471-2105-5-113 PMC517706

[ece34935-bib-0023] Fairbairn, D. J. (1997). Allometry for sexual size dimorphism: Pattern and process in the coevolution of body size in males and females. Annual Review of Ecology and Systematics, 28, 659–687. 10.1146/annurev.ecolsys.28.1.659

[ece34935-bib-0024] Fairbairn, D. J. , Blanckenhorn, W. U. , & Székely, T. (2007). Sex, size, and gender roles: Evolutionary studies of sexual size dimorphism. Oxford, UK: Oxford University Press.

[ece34935-bib-0025] Felsenstein, J. (1985). Phylogenies and the Comparative Method. American Naturalist, 125, 3–147. 10.1086/703055 31094602

[ece34935-bib-0026] Foellmer, M. W. , & Moya‐Larano, J. (2007). Sexual size dimorphism in spiders: Patterns and processes In Fairbairn D., Blanckenhorn W. U., & Székely T. (Eds.), Sex, size and gender roles: Evolutionary studies of sexual size dimorphism (pp. 71–81). Oxford, UK: Oxford University Press.

[ece34935-bib-0027] Françoso, E. , & Arias, M. C. (2013). Cytochrome c oxidase I primers for corbiculate bees: DNA barcode and mini‐barcode. Molecular Ecology Resources, 13, 844–850. 23848578 10.1111/1755-0998.12135

[ece34935-bib-0028] Greenleaf, S. S. , Williams, N. M. , Winfree, R. , & Kremen, C. (2007). Bee foraging ranges and their relationship to body size. Oecologia, 153, 589–596. 10.1007/s00442-007-0752-9 17483965

[ece34935-bib-0029] Halcroft, M. T. , Dollin, A. , Francoy, T. M. , King, J. E. , Riegler, M. , Haigh, A. M. , & Spooner‐Hart, R. N. (2016). Delimiting the species within the genus *Austroplebeia*, an Australian stingless bee, using multiple methodologies. Apidologie, 47, 76–89. 10.1007/s13592-015-0377-7

[ece34935-bib-0030] Harvey, P. H. , & Pagel, M. D. (1991). The comparative method in evolutionary biology. Oxford, UK: Oxford University Press.

[ece34935-bib-0031] Imperatriz‐Fonseca, V. L. , & Zucchi, R. (1995). Virgin queens in stingless bee (Apidae, Meliponinae) colonies: A review. Apidologie, 26, 231–244. 10.1051/apido:19950305

[ece34935-bib-0032] Jarau, S. , van Veen, J. W. , Twele, R. , Reichle, C. , Gonzales, E. H. , Aguilar, I. , … Ayasse, M. (2010). Workers make the queens in *Melipona* bees: Identification of geraniol as a caste determining compound from labial glands of nurse bees. Journal of Chemical Ecology, 36, 565–569. 10.1007/s10886-010-9793-3 20431925

[ece34935-bib-0033] Kerr, W. E. (1948). Estudos sôbre o gênero *Melipona* . Anais Da E.S.A. ”luis De Queiroz”, 5, 181–275.

[ece34935-bib-0034] Kovacs, J. L. , Hoffman, E. A. , Marriner, S. M. , & Goodisman, M. A. D. (2010). Detecting selection on morphological traits in social insect castes: The case of the social wasp *Vespula maculifrons* . Biological Journal of the Linnean Society, 101, 93–102. 10.1111/j.1095-8312.2010.01495.x

[ece34935-bib-0035] Kratochvíl, L. , & Frynta, D. (2007).Phylogenetic analysis of sexual dimorphism in eye‐lid geckos (Eublepharidae): The effects of male combat, courtship behavior, egg size, and body size In Fairbairn D. J., Blanckenhorn W. U., & Székely T. (Eds.), Sex, size and gender roles: Evolutionary studies of sexual size dimorphism (pp. 154–162). New York, NY: Oxford University Press.

[ece34935-bib-0036] Lanfear, R. , Calcott, B. , Kainer, D. , Mayer, C. , & Stamatakis, A. (2014). Selecting optimal partitioning schemes for phylogenomic datasets. BMC Evolutionary Biology, 14, 82 10.1186/1471-2148-14-82 24742000 PMC4012149

[ece34935-bib-0037] Linksvayer, T. A. , & Wade, M. J. (2009). Genes with social effects are expected to harbor more sequence variation within and between species. Evolution, 63, 1685–1696. 10.1111/j.1558-5646.2009.00670.x 19245396 PMC3151725

[ece34935-bib-0038] Lovich, J. E. , & Gibbons, J. W. (1992). A review of techniques for quantifying sexual size dimorphism. Growth, Development, and Aging, 56, 269–281. 1487365

[ece34935-bib-0039] Macías‐Macías, J. O. , & Quezada‐Euán, J. J. G. (2015). Stingless bees in a temperate climate: Oviposition behavior and duration of ontogenic development stages in *Melipona colimana* (Hymenoptera: Meliponini). Journal of Apicultural Research, 54, 255–259.

[ece34935-bib-0040] Maddison, W. P. , & Maddison, D. R. (2015). *Mesquite: * *A* * modular system for evolutionary analysis* . Version 1.05. Retrieved from http://mesquiteproject.org

[ece34935-bib-0041] May‐Itza, W. , Quezada‐Euán, J. J. G. , Medina, L. A. M. , Enríquez, E. , & De la Rúa, P. (2010). Morphometric and genetic differentiation in isolated populations of the endangered Mesoamerican stingless bee *Melipona yucatanica* (Hymenoptera: Apoidea) suggest the existence of a two species complex. Conservation Genetics, 11, 2079–2084. 10.1007/s10592-010-0087-7

[ece34935-bib-0042] Medina, R. G. , Fairbairn, D. J. , Bustillos, A. , Moo‐Valle, H. , Medina, S. , & Quezada‐Euán, J. J. G. (2016). Variable patterns of intraspecific sexual size dimorphism and allometry in three species of eusocial corbiculate bees. Insectes Sociaux, 63, 493–500. 10.1007/s00040-016-0491-1

[ece34935-bib-0043] Michener, C. D. (1974). The social behavior of the bees: A comparative study. Cambridge, MA: Belknap Press of Harvard University Press.

[ece34935-bib-0044] Miller, M. A. , Pfeiffer, W. , & Schwartz, T. (2010). Creating the CIPRES science gateway for inference of large phylogenetic trees. New Orleans, LA: IEEE.

[ece34935-bib-0045] Moo‐Valle, H. , Quezada‐Euán, J. J. , Canto‐Martín, J. , & González‐Acereto, J. A. (2004). Caste ontogeny and the distribution of reproductive cells on the combs of *Melipona beecheii* (Apidae: Meliponini). Apidologie, 35, 587–594.

[ece34935-bib-0046] Morales, A. (2018) *Efecto Del Alimento Larval Sobre El Tiempo de Desarrollo, Tamaño corporal y potencial reproductivo en reinas de Scaptotrigona pectoralis (Hymenoptera: Meliponini) Criadas in Vitro. MSc Thesis* . Merida, Yucatan: Universidad Autónoma de Yucatán.

[ece34935-bib-0047] Oldroyd, B. P. , & Wongsiri, S. (2006). Asian honey bees: biology, conservation and human interactions. Harvard, USA: Harvard University Press.

[ece34935-bib-0048] Orme, D. , Freckleton, R. , Thomas, G. , Petzoldt, T. , Fritz, S. , Isaac, N. , & Pearse, W. (2013) *caper: Comparative Analyses of Phylogenetics and Evolution in R* . R package (version 0.5.2). Retrieved from http://cran.r-project.org/package=caper

[ece34935-bib-0049] Paradis, E. , Claude, J. , & Strimmer, K. (2004). APE: Analyses of phylogenetics and evolution in R language. Bioinformatics, 20, 289–290. 10.1093/bioinformatics/btg412 14734327

[ece34935-bib-0050] Paxton, R. J. (2005). Male mating behaviour and mating systems of bees: An overview. Apidologie, 36, 145–156.

[ece34935-bib-0051] Quezada‐Euán, J. J. G. (2018). Stingless bees of Mexico: The biology, management and conservation of an ancient heritage. New York, NY: Springer Nature.

[ece34935-bib-0052] Quezada‐Euán, J. J. G. , López‐Velasco, A. , Pérez‐Balam, J. , Moo‐Valle, H. , Velazquez‐Madrazo, A. , & Paxton, R. J. (2011). Body size differs in workers produced across time and is associated with variation in the quantity and composition of larval food in *Nannotrigona perilampoides* (Hymenoptera, Meliponini). Insectes Sociaux, 58, 31–38. 10.1007/s00040-010-0113-2

[ece34935-bib-0053] R Core Team . (2015). R: A Language and environment for statistical computing (Vol. 1, pp. 409). Vienna, Austria: R Foundation for Statistical Computing.

[ece34935-bib-0054] R Development Core Team (2013). R: A language and environment for statistical computing. Vienna, Austria: R Foundation for Statistical Computing.

[ece34935-bib-0055] Rambaut, A. , Drummond, A. J. , Xie, D. , Baele, G. , & Suchard, M. A. (2018). Posterior summarization in bayesian phylogenetics using Tracer 1.7 (ed E Susko). Systematic Biology, 67, 901–904. 10.1093/sysbio/syy032 29718447 PMC6101584

[ece34935-bib-0056] Ramírez, S. R. , Nieh, J. C. , Quental, T. B. , Roubik, D. W. , Imperatriz‐Fonseca, V. L. , & Pierce, N. E. (2010). A molecular phylogeny of the stingless bee genus *Melipona *(Hymenoptera: Apidae). Molecular Phylogenetics and Evolution, 56, 519–525. 10.1016/j.ympev.2010.04.026 20433931

[ece34935-bib-0057] Rasmussen, C. , & Cameron, S. A. (2010). Global stingless bee phylogeny supports ancient divergence, vicariance, and long distance dispersal. Biological Journal of the Linnean Society, 99, 206–232. 10.1111/j.1095-8312.2009.01341.x

[ece34935-bib-0058] Ratnieks, F. (2001). Heirs and spares: Caste conflict and excess queen production in *Melipona* bees. Behavioral Ecology and Sociobiology, 50, 467–473. 10.1007/s002650100388

[ece34935-bib-0059] Ratnieks, F. L. W. , & Wenseleers, T. (2005). Policing insect societies. Science, 307, 54–56. 15637260 10.1126/science.1106934

[ece34935-bib-0060] Rensch, B. (1950). Dieabhangigkeit der relative Sexualdifferenz von der K¨orpergrofse. Bonner Zoologische Beitr¨age, 1, 58–69.

[ece34935-bib-0061] Revell, L. J. (2012). phytools: An R package for phylogenetic comparative biology (and other things). Methods in Ecology and Evolution, 3, 217–223. 10.1111/j.2041-210X.2011.00169.x

[ece34935-bib-0062] Revell, L. J. (2013). Two new graphical methods for mapping trait evolution on phylogenies. Methods in Ecology and Evolution, 4, 754–759. 10.1111/2041-210X.12066

[ece34935-bib-0063] Revell, L. J. (2014). Modern phylogenetic comparative methods and their application in evolutionary biology InGaramszegi L. Z. (Ed.), Modern phylogenetic comparative methods and their application in evolutionary biology (pp. 77–103). Berlin: Springer.

[ece34935-bib-0064] Ribeiro, M. d. F. , Wenseleers, T. , Santos Filho, P. d. S. , & Alves, D. d. A. (2006). Miniature queens in stingless bees: Basic facts and evolutionary hypotheses. Apidologie, 37, 191–206. 10.1051/apido:2006023

[ece34935-bib-0065] Ronquist, F. , Teslenko, M. , van der Mark, P. , Ayres, D. L. , Darling, A. , Höhna, S. , … Huelsenbeck, J. P. (2012). Mrbayes 3.2: Efficient bayesian phylogenetic inference and model choice across a large model space. Systematic Biology, 61, 539–542. 10.1093/sysbio/sys029 22357727 PMC3329765

[ece34935-bib-0066] Ruiz, C. , May-Itza, W. de J. , Quezada-Euán, J. J. G. , & De la Rúa, P. (2013). Presence of nuclear copies of mitochondrial origin (NUMTs) in two related species of stingless bee genus *Melipona* (Hymenoptera: Meliponini). Journal of Zoological Systematics and Evolutionary Research, 51, 107–113.

[ece34935-bib-0067] Sakagami, S. F. (1982). Stingless bees In Hermann H. R. (Ed.) Social insects (Vol. III, pp. 361–423). London, UK: Academic Press.

[ece34935-bib-0068] Schwarz, H. F. (1932). The genus *Melipona*, the type genus of the Meliponidae or stingless bees. Bulletin of the American Museum of Natural History, 63, 1–460.

[ece34935-bib-0069] Schwarz, H. F. (1948). Stingless bees (Meliponidae) of the Western hemisphere. Bulletin of the American Museum of Natural History, 90, 1–536.

[ece34935-bib-0070] Serrano‐Meneses, M. A. , Cordoba‐Aguilar, A. , Azpilicueta‐Amorín, M. , González‐Soriano, E. , & Székely, T. (2008). Sexual selection, sexual size dimorphism and Rensch’s rule in Odonata. Journal of Evolutionary Biology, 21, 1259–1273. 10.1111/j.1420-9101.2008.01567.x 18636976

[ece34935-bib-0071] Shreeves, G. , & Field, J. (2008). Parental care and sexual size dimorphism in wasps and bees. Behavioral Ecology and Sociobiology, 62, 843–852. 10.1007/s00265-007-0510-3

[ece34935-bib-0072] Stillwell, R. C. , Blanckenhorn, W. U. , Teder, T. , Davidowitz, G. , & Fox, C. W. (2010). Sex differences in phenotypic plasticity affect variation in sexual size dimorphism in insects: From physiology to evolution. Annual Review of Entomology, 55, 227–245. 10.1146/annurev-ento-112408-085500 PMC4760685 19728836

[ece34935-bib-0073] Stubblefield, J. W. , & Seger, J. (1994). Sexual dimorphism in the ymenoptera In Short R. V., & Balaban E. (Eds.), The differences between the sexes (pp. 71–104). Cambridge, U.K: Cambridge University Press.

[ece34935-bib-0074] Sung, I.‐H. , Yamane, S. , Ho, K.‐K. , Wu, W.‐J. , & Chen, Y.‐W. (2004). Morphological caste and sex differences in the Taiwanese stingless bee *Trigona ventralis hoozana* (Hymenoptera: Apidae). Entomological Science, 7, 263–269. 10.1111/j.1479-8298.2004.00072.x

[ece34935-bib-0075] Tammaru, T. , Esperk, T. , Ivanov, V. , & Teder, T. (2010). Proximate sources of sexual size dimorphism in insects: Locating constraints on larval growth schedules. Evolutionary Ecology, 24, 161–175. 10.1007/s10682-009-9297-1

[ece34935-bib-0076] Teder, T. (2014). Sexual size dimorphism requires a corresponding sex difference in development time: A meta‐analysis in insects. Functional Ecology, 28, 479–486. 10.1111/1365-2435.12172

[ece34935-bib-0077] Teder, T. , & Tammaru, T. (2005). Sexual size dimorphism within species increases with body size in insects. Oikos, 108, 321–334. 10.1111/j.0030-1299.2005.13609.x

[ece34935-bib-0078] Warton, D. I. , Duursma, R. A. , Falster, D. S. , & Taskinen, S. (2012). smatr 3‐ an R package for estimation and inference about allometric lines. Methods in Ecology and Evolution, 3, 257–259. 10.1111/j.2041-210X.2011.00153.x

[ece34935-bib-0079] Webb, T. J. , & Freckleton, R. P. (2007). Only half right: Species with female‐biased sexual size dimorphism consistently break Rensch’s rule. PLoS ONE, 2(9), e897 10.1371/journal.pone.0000897 17878932 PMC1964802

[ece34935-bib-0080] Wenseleers, T. , Hart, A. G. , Ratnieks, F. L. W. , & Quezada‐Euán, J. J. G. (2004). Queen execution and caste conflict in the stingless bee *Melipona beecheii* . Ethology, 110, 725–736. 10.1111/j.1439-0310.2004.01008.x

[ece34935-bib-0081] Wilson, E. O. (1971). The insect societies. Cambridge, MA: Harvard University Press.

