# Supplementary figures and images for: Patterns of sexual size dimorphism in stingless bees: Testing Rensch’s rule and potential causes in highly eusocial bees (Hymenoptera: Apidae, Meliponini)
Source: Ecol Evol. 2019 Feb 5;9(5):2688–98. doi: 10.1002/ece3.4935 (PMC6405504; doi:10.1002/ece3.4935)

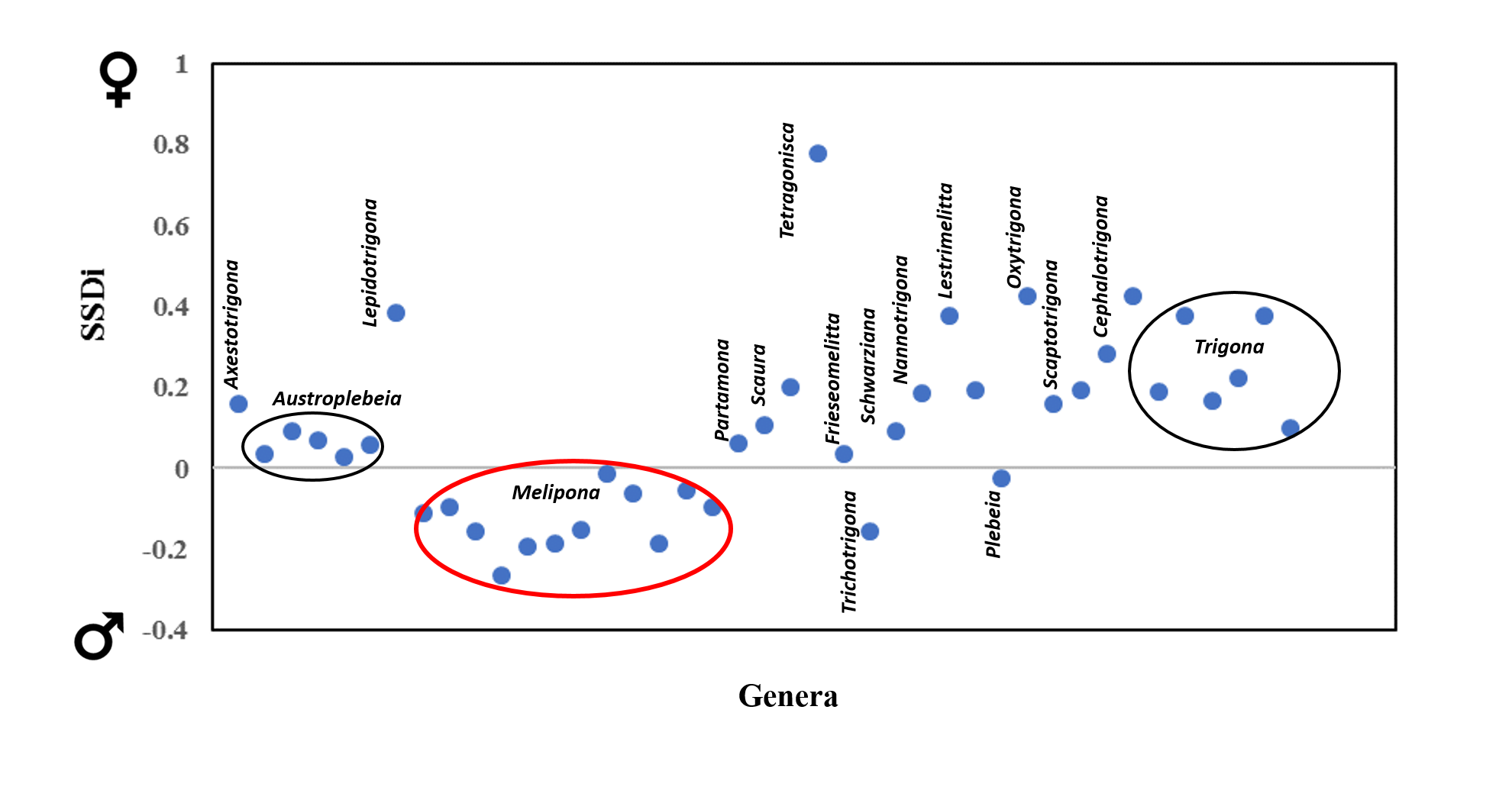

Supplement: Supplementary file 1 — FigS1 [file ECE3-9-2688-s001.png]
